# Aspects of prophylactic treatment of hemophilia

**DOI:** 10.1186/s12959-016-0103-3

**Published:** 2016-10-04

**Authors:** Rolf Ljung

**Affiliations:** 1 Department of Clinical Sciences Lund-Paediatrics, Lund University, Lund, Sweden; 2 Department of Paediatrics and Malmö Centre for Thrombosis and Haemostasis, Skåne University Hospital, Malmö, Sweden

**Keywords:** Hemophilia A, Hemophilia B, Factor VIII, Factor IX, Prophylaxis

## Abstract

Retrospective and prospective studies unambiguously show that prophylactic treatment of severe hemophilia A or B should be started as primary prophylaxis at 1–2 years’ of age and ideally before the first joint bleed. The dose and dose frequency should be individually tailored depending on the goal of treatment, venous access and the bleeding phenotype. The circumstances during the first exposures of factor VIII concentrates in hemophilia A may have an impact on the risk of developing inhibitors. Enhanced half-life products, in particular in hemophilia B, will facilitate treatment in patients with difficult venous access but also in achieving a higher trough level. Evidence accumulate that prophylactic treatment is beneficial also in adults and in patients with inhibitors.

## Background

According to a joint statement made by the World Health Organization (WHO) and the World Federation of Hemophilia (WFH), initiating prophylactic treatment at an early age is considered to be the optimal form of therapy for a child with severe hemophilia [1–3]. However, at that time no definition was given of ‘prophylactic therapy’ for hemophilia. According to a suggested definition from the Scientific and Standardization Committee (SSC) of the International Society on Thrombosis and Haemostasis (ISTH) [4], primary prophylaxis is a continuous therapy starting after the first joint bleed and before the age of 3 years. Alternatively, primary prophylaxis can be a continuous treatment started before the age of 3 years in a patient without any previous joint bleed (i.e. initiated based solely on age). Secondary prophylaxis can either be continuous long-term treatment started after two or more joint bleeds, or after the age of 3 years; however, secondary prophylaxis can also be an intermittent periodic prophylactic treatment.

Prophylactic treatment using various regiments has been practiced for many decades in Sweden and The Netherlands and have clearly demonstrated the benefit of prophylaxis in several retrospective and observational studies. However, the randomized controlled US study of Manco-Johnson et al. [5] gave the final proof of the concept of prophylactic treatment of children. Sixty boys were randomly assigned to prophylaxis (*n* = 32) or on-demand therapy (*n* = 32). The boys in the prophylactic group had a median of 1.2 hemorrhages versus 17.1 per year in the on demand group and had, respectively, a mean of 0.6 joint bleeds per year compared to 4.9. However, at the same time they consumed three times as much factor VIII (FVIII) although there was a tendency to increased consumption over time in the on demand group.

In the literature one may find a variety of opinions on when to start treatment prophylactic treatment, the dose and dosage interval [6]. Most children in the world do not have access to prophylactic treatment, the main obstacles being the cost of FVIII/IX concentrates and, at least in the youngest age group, venous access.

Do children with hemophilia B have the bleeding phenotype as those with hemophilia A and thus the same need for prophylactic therapy? In a recent paper, based on the PedNet Registry, Clausen et al. [7] found no difference in the bleeding phenotype in early age of severe or moderate hemophilia A and B that could motivate a different approach to prophylactic treatment in hemophilia B compared to hemophilia A.

## Review

### When should prophylactic treatment begin?

The experience from Sweden (Astermark et al. [8]) suggests that prophylaxis should be started early. In that study, the frequency of joint bleeds and joint scores were assessed in 121 patients with severe hemophilia and patients were stratified in and three subgroups: (1) children who had begun prophylaxis before age 3 years (*n* = 75), (2) children between age 3–5 years (*n* = 31), and (3) those who had begun between age 6–9 years (*n* = 15). The group starting prophylaxis before two years of age had a significantly higher proportion of children with no joint damage compared to the other groups, demonstrating the benefit of starting prophylactic treatment at an early age.

The objectives of the Canadian ‘Hemophilia Dose Escalation Prophylaxis Trial’, was to avoid a central venous access device and to individualize treatment according to the observed bleeding phenotype [9]. Children were initially treated once-weekly with 50 IU/kg and the frequency of injections was escalated in a stepwise fashion if unacceptable bleeding occurred. Several bleeds during a 3-month period were accepted before escalating and despite a rather good outcome on physical examination, evaluation by magnetic resonance imaging (MRI) of 24 subjects revealed osteochondral changes in 50 % of the subjects and 9 % of the joints. For the future it will probably be of clinical significance that soft tissue changes were detected in, 75 % of ankles (12/16), 19 % of elbows (6/32) and 12 % of knees (2/17) that had been reported as “bleed free” [10].

The benefits of prophylactic treatment are usually evaluated by different joint outcome measurers. The risk of intracranial hemorrhage (ICH) after the neonatal period is 20–50 times higher in a person with hemophilia on demand treatment compared to a non-hemophiliac [11–14]. This is a fact that should be taken into account when discussing in particular the dose interval in prophylactic treatment of hemophilia.

Once hemophilic arthropathy has begun it may, at least in some susceptible individuals, progress despite adequate therapy [15] and higher doses are needed to keep the patient bleed free. This is another argument for primary prophylaxis to be started before the first joint bleed.

### Which dose and dose interval?

The prophylactic regiment used is dependent on the objective of treatment, the economic resources available and, in particular in young children, venous access. A vision for the future of a trough level of 15 % has been expressed by the World Federation of Hemophilia (WFH) [16]. The outcome related to start of treatment and the dose used, was studied in a long-term follow-up [17] of the prophylactic regimens for patients with severe hemophilia (FVIII/IX < 1 IU/dL) in The Netherlands and Sweden, the two countries with the longest clinical experience of prophylaxis but with slightly different approach to age at start of treatment and dosing. The patients were born between 1970 and 1994 and had at follow up a median age of approximately 25 years. Early start of treatment and higher doses gave fewer joint bleeds but small differences were found at this age in clinical outcome parameters. However, the cost for the high dose regimen was twice. The life-long outcome, and thus if the higher costs of a “high-dose” model is justified, are presently unknown.

Several studies suggest that there is a subgroup of patients who are more susceptible to synovitis and progressive arthropathy after a joint bleed. The existence of subclinical bleeds was suggested several years ago from Sweden [18, 19], and has been suggested as cause of advanced joint changes found on MRI in patients reporting no or very few joint bleeds [5, 20]. The problem is that we have little knowledge how to define this subgroup who are most susceptible to developing arthropathy.

### Individualized prophylactic regimens

It is obvious that the prophylactic dose and dosage interval should be tailored individually, depending on the clinical aim of treatment, the bleeding phenotype, the patient’s daily activities, venous access and cost-efficacy being the most important factors. The time spent with a FVIII level below 1 IU/dL in patients with hemophilia A is associated with both the total number of bleeds and the number of joint bleeds [21]. Pharmacokinetic (PK) measurement is a useful tool to guide and monitor treatment. It can also be used to educate patients, for example, that a dose escalation on Friday to cover the whole weekend would require very high doses of FVIII [22]. Web-based user-friendly instruments have been developed that will facilitate PK evaluation of changes in dose and dose interval in the individual patient. This will be most useful when introducing extended half-life concentrates [23].

### Prophylaxis and inhibitors

Several studies in hemophilia A have shown that patients, starting the first 20 exposures as prophylactic treatment, compared to on demand treatment due to a bleed, have a decreased risk to develop inhibitors [24–27]. The RODIN study [28], which is a prospective observational study (*n* = 574), modified these finding since it did not show any difference in the rate of inhibitors during the first 20 exposure days between prophylaxis and on-demand treatment. However, after the first 20 exposure days, prophylaxis was associated with a HR (hazard ratio) of 0.68; 95 % CI: 0.47–0.99, *i.e.* a protective effect and the inhibitors that developed were mainly low titer. It may indicate that some patients at high risk to develop inhibitors (type of mutation, family history of inhibitors and other genetic risk factors) will develop inhibitors despite the mode of treatment they receive during the first 20 exposure days. However, in patients with low genetic risk it seems that it may be possible to reduce the risk of inhibitors by introducing regular prophylactic treatment.

In addition, one should probably in severe hemophilia A during the first 20 exposure days, avoid ‘immunological danger signals’ such as inflammation/infection/vaccination, intensive treatment with high doses on consecutive days for example during surgical procedures.

Inhibitors are less frequent in hemophilia B and we do not know if the risk factors for inhibitors found in hemophilia A are applicable also to hemophilia B.

### Prophylaxis in adults

Opinions vary on the need of prophylactic treatment in adulthood. The SPINART study [29] is the first prospective, controlled, randomized study comparing routine prophylaxis with on-demand treatment in adults with severe hemophilia A. The median number of total bleeding episodes and total bleeding episodes per year were significantly lower with prophylaxis than with on-demand treatment (total: 0 versus 54.5; total per year: 0 versus 27.9; both *P* < 0.0001). A Swedish retrospective study on prophylaxis in adults showed that only 36 % of all patients experienced a joint bleed in a 3-year period [30]. In comparison, patients treated with on-demand therapy are likely to have 30–35 joint bleeds per year [31].

### Prophylaxis and enhanced half-life (EHL) products

Novel longer-acting products are now being introduced or are in the pipeline from several manufacturers [32]. The half-life is only moderately prolonged in recombinant FVIII (1.5-fold) but significantly prolonged in recombinant FIX (2.4-4.8-fold). Three EHL-rFIX products have completed phase 3 clinical studies [33–35]. Different principles have been used to prolong action in these three concentrates, fusion with albumin respectively Fc portion of IgG or addition of a PEG (polyethylenglycole). The experiences have been good when using EHL- FIX for prophylaxis with a dosing frequency between 7 and 14 days.

Fc fusion and PEGylation technologies have also been used to produce EHL-rFVIII. There are 4 EHL-FVIII, one Fc-fusion and three pegylated products of which some licensed in some countries and some are under development. The pegylated products have used different strategies concerning the pegylation and also attach PEGs of different sizes, 60, 40 and 2 × 20 kDa [36]. One of them uses full-length rFVIII while the other three are B-domain deleted rFVIII. The half-life extension of rFVIII products is in the range of 1.4–1.6 fold, considerably shorter than EHL-FIX. Not much data are published yet on PUPs and we do not know the frequency of inhibitors that will develop in PUPs.

In patients with difficult venous access, products with an enhanced half-life will be useful and improve compliance. However, the experience we have gained from the conventional concentrates may not be applicable to the longer-acting ones without some considerations. Most patients will, with less frequent injections and without increasing the consumption of concentrate, probably spend a longer time under a certain concentration and have fewer peaks, which may increase the risk for breakthrough bleeds, subclinical/micro-bleeds and change our view on allowance of sports activities. On the other hand, depending on costs, the availability of the longer-acting products opens up a scenario that persons with hemophilia with today’s frequency of injections may have a trough level equivalent to that of a patient with mild hemophilia, which would be a paradigm shift.

### Prophylactic treatment of patients with inhibitors

Patients with hemophilia A or B who have developed inhibitors may be treated prophylactically with increased doses of FVIII/IX if the inhibitor titer is very low (max. 1–2 BU). Bypassing agents, activated prothrombin complex concentrates (aPCC; FEIBA® Baxalta) and the recombinant activated factor VII (rFVIIa; Novo- Seven®, Novo Nordisk), have been used to treat bleeding episodes in patients with inhibitors. However, the benefits of prophylaxis with bypassing agents are not as efficacious as in non-inhibitor patients, but they have been shown to be effective in three prospective, randomized trials. Two studies evaluated aPCC, Pro–FEIBA and PROOF studies [37, 38], with a 60–72 % reduction of bleeding episodes compared to on-demand therapy and one evaluated rFVIIa [39] with up to 60 % reduction of bleeding episodes compared to the pre-prophylactic period. Prophylactic treatment of patients with inhibitors may be considered in the pre-ITI period, during ITI or in cases with failed ITI. The problems are the short half-life of the by-passing agents, the lower efficacy compared to FVIII/IX and the considerable costs which have limited its use. A panel of Spanish hematologists has recently made a systematic review of the literature with the objective to develop consensus based guidelines [40].

## Conclusions

Initiating treatment at an early age is the optimal form of therapy for a child with hemophilia A or B. The dose and dosage interval of prophylactic treatment are dependent on the goal of therapy, the available economic resources and venous access [41]. The number of joint bleeds should not be the only outcome parameter, the risk of subclinical micro-bleeds as well as the risk for intracranial bleeds need to be considered [41]. Pharmacokinetics is a useful tool to monitor treatment. There is evidence that prophylaxis should be extended into adulthood [41].

## Abbreviations

EHL: Enhanced half-life products
FIX: Factor IX
FVIII: Factor VIII
